# Endocardite Infecciosa: Ainda mais Desafios que Certezas

**DOI:** 10.36660/abc.20200798

**Published:** 2022-05-04

**Authors:** Catarina Sousa, Fausto J. Pinto

**Affiliations:** 1 Centro Cardiovascular Universidade de Lisboa Faculdade de Medicina Universidade de Lisboa Lisboa Portugal Centro Cardiovascular Universidade de Lisboa (CCUL), Faculdade de Medicina, Universidade de Lisboa, Lisboa – Portugal; 2 Serviço de Cardiologia Centro Hospitalar Barreiro Montijo Barreiro Portugal Serviço de Cardiologia, Centro Hospitalar Barreiro Montijo (CHBM), Barreiro – Portugal; 3 Departamento Coração e Vasos Centro Hospitalar e Universitário Lisboa Norte Lisboa Portugal Departamento Coração e Vasos, Centro Hospitalar e Universitário Lisboa Norte (CHULN), Lisboa – Portugal

**Keywords:** Palavras-chave: Endocardite Infecciosa, Epidemiologia, Diagnóstico, Profilaxia

## Abstract

Após catorze décadas de evolução médica e tecnológica, a endocardite infeciosa continua a desafiar médicos no seu diagnóstico e manejo diário. O aumento da incidência, alterações demográficas (afetando pacientes mais idosos), microbiologia com taxas de infeção por *Staphylococcus* mais elevadas, com complicações graves ainda frequentes e uma mortalidade substancial tornam a endocardite uma doença muito complexa. Apesar de tudo, a inovação no seu diagnóstico, nomeadamente na área da microbiologia e imagem, e a melhoria nos cuidados intensivos e na cirurgia cardíaca (quanto às técnicas, materiais usados e momento de intervenção) podem ter um impacto no seu prognóstico. Os desafios persistem, incluindo repensar a profilaxia, melhorar os critérios de diagnóstico incluindo a endocardite com culturas negativas e endocardite de prótese valvar, o *timing* para a intervenção cirúrgica, e sua realização ou não na presença de acidente vascular cerebral isquêmico e em usuários de drogas intravenosas. Uma estratégia combinada na endocardite infeciosa é fundamental, incluindo decisões e protocolos clínicos avançados, um manejo multidisciplinar, organização e políticas de saúde que culminem em melhores resultados para os nossos pacientes.

“Vale a pena, de tempos e tempos, reavaliar nosso

conhecimento sobre uma doença específica, para

verificar exatamente nossa posição em relação a ela e

a que conclusões os fatos acumulados até o momento

apontam, e para determinar a que direção deveremos

olhar visando investigações produtivas no futuro”.

*William Osler (1885)*

## Epidemiologia

### Incidência e aspectos demográficos

A incidência da endocardite infecciosa (EI) varia entre 3 e 15 casos por 100 000 em estudos populacionais.^1^ Tal variação provavelmente esteja relacionada a vários fatores: critérios para definição dos casos (caso definitivo, caso possível, inclusão de EI com cultura negativa), diferentes fontes de caso ou o período temporal analisado tendo como referência a publicação das diretrizes. Nesta análise, não incluímos estudos observacionais unicêntricos ou multicêntricos com alto risco de viés de seleção que poderiam, então, subestimar a real incidência da doença. Observou-se uma predominância do sexo masculino, com uma razão de homens:mulheres entre 0,96 e 2.8. Tal fato também é observado em grandes registros internacionais, tais como o estudo ICE,^4^ o registro GAMES na Espanha,^5^ ou o estudo EURO-ENDO recentemente publicado.^6^

Na maioria das séries populacionais e registros internacionais, os pacientes mais velhos são os geralmente mais afetados, com idade mediana entre 50 (mais para fim da década) e 60 anos ( Tabela 1 ). Ainda, a incidência aumenta com a idade.^7^

**Tabela 1:** – Estudos populacionais sobre incidência, aspectos demográficos e desfechos da endocardite bacteriana

Autores/estudo	País	Tamanho amostral (Fonte)	Período de tempo	Fontes dos casos	Número de casos	Incidência/100 000	Faixa etária	Homens/Mulheres	Cultura negativa (%)	Desfechos: cirurgia de válvula e mortalidade
Fonager et al.^1^	Dinamarca	5,2 milhões	1980-1997	Registro nacional da Dinamarca	3351	Análise por sexo: Homens 4 - 6 Mulheres 3 - 4	60,4	1,32	ND	Taxa de letalidade em 30 dias 23%
Tleyjeh et al.^2^	Estados Unidos (Olmsted)	1970 - 51 000 2000 - 90 000	1970-2000	Projeto de epidemiologia Rochester	107	5,0-7,0	61,5 (18,8-90,6)	2,7	1	Cirurgia de válvula 15% Mortalidade em seis meses 14-33%
Sy et al.^7^	Austrália (Nova Gales do Sul)	6,6 milhões	2000-2006	Banco de dados estadual	1536	4,7	62 (42-75)	1,8	15	Cirurgia de válvula 20% Mortalidade hospitalar 14% Mortalidade em seis meses 18%
Fedeli et al.^8^	Itália (Veneto)	4,8 milhões	2000-2008	Arquivos eletrônicos dos registros de alta hospitalar	1863	4,4	68	1,4 - 2	ND	Cirurgia de válvula 23% Mortalidade hospitalar 14%
Selton-Suty et al.^108^	França (grande Paris, Lorraine, Rhône-Alpes, Franco- Condado, Marne, Ille-et-Vilaine, eLanguedoc-Roussillon)	15,3 milhões	2008	Formulários de relato de caso enviados a médicos envolvidos no tratamento de endocardite infecciosa	497	3,2	62,3	2,8	5	Cirurgia de válvula 45% Mortalidade hospitalar 23%
Bikdeli et al.^109^	EUA	NA (pacientes internados >65 anos de idade)	1999-2010	Serviços do Medicare & Medicaid Arquivos padrões de internação de pacientes usuários do Medicare	262 658	7,2	79	0,7-0,8	ND	Mortalidade hospitalar 9-11% Mortalidade em 30 dias – 14-16,5% Mortalidade em um ano – 32,6-36,2%
Duval et al.^9^	França (grande Paris, Lorraine, e Ródano-Alpes)	11 milhões	Inquérito de um ano (1991, 1999 e 2008)	Formulários de relato de caso enviados a médicos envolvidos no tratamento de endocardite infecciosa	1991 - 323 1999 – 331 2008 - 339	1991 – 3,5 1999 – 3,3 2008 – 3,2	1991 – 57,9 1999 – 59,8 2008 – 61,6	1991 – 1,9 1999 – 2,3 2008 – 2,9	ND	Cirurgia valvar (1991 – 31,3%; 1999 – 50,2%; 2008 – 49,6%) Mortalidade hospitalar (1991 – 20,7%; 1999 – 15,4%; 2008 – 21,2%)
Ternhag et al.^110^	Suécia		1997-2007	Registro de alta hospitalar do Swedish Hospital	7603	7,7	65,7	1,45	ND	Mortalidade de 30 dias – 10,4% Taxa de mortalidade 1-5 anos 14,7%
Dayer et al.^111^	Inglaterra	ND	2000-2013	Dados do *Secondary Uses Service*	19804	2,7	ND	ND	ND	ND
Pant et al.^112^	EUA	ND	2000-2011	Banco de dados nacional de pacientes hospitalizados	457052	11-15	ND	ND	ND	ND
Erichsen et al.^10^	Dinamarca	ND	1994–2011	Registro nacional da Dinamarca	5486	7,55 (2009–2011)	63	1,8	ND	ND
Keller et al.^62^	Alemanha	80 milhões	2005-2014	Banco de dados nacional do Departamento de Estatística da Alemanha	94364	11,6	ND	ND	ND	Mortalidade hospitalar 17%
Toyoda et al.^11^	EUA (Califórnia e estado de Nova Iorque)	ND	1998-2013	Banco de dados estadual cooperativo de planejamento e pesquisa (Nova Iorque); Banco de dados estadual do serviço de desenvolvimento e planejamento da saúde (Califórnia)	75 829	7,6 a 9,3	62,3	1,2	25	Cirurgia de válvula - 17-13% Mortalidade em 90 dias– 24%
Van den Brink et al. ^113^	Países baixos	ND	2005-2011	Autoridade em saúde dos Países Baixos	5213	2005-3.0 2011- 6.3	66,4	2,3	9.3	Mortalidade por todas as causas 36,1%
Cresti et al.^3^	Itália (Grosseto)	217 778	1998-2014	Dados do sistema de saúde	170	4.6	69,5	1,54	19	Cirurgia de válvula – 46% Mortalidade hospitalar 25% Mortalidade em um ano 32%
Ahtela et al.^114^	Finlândia	ND	2005-2014	Dados do sistema de saúde ( *Care Register for Health Care* )	2611	6.33	60,0	2,1	ND	Mortalidade em 30 dias– 11,3%
Olmos et al.^97^	Espanha	ND	2003-2014	Sistema nacional de saúde da Espanha	16867	2,7 em 2003 3,5 em 2014 por 100 000 pessoas/ano	ND	2	ND	Mortalidade hospitalar 20,4%
Shah et al.^65^	Escócia	ND	1990-2014	Registro nacional de internação hospitalar Registro nacional de microbiologia	7638	5,3 em 1990	65	0,96	58	Desfecho em 30 dias: Mortalidade por todas as causas– 9,6%-17,3%% Cirurgia de válvula – 3,8-6,3% Desfecho em um ano: mortalidade por todas as causas– 27-33,3% Cirurgia de válvula– 9,2-15,5%

*ND: não disponível.*

Finalmente, uma metanálise publicada em 2013^12^ que incluiu 160 estudos de todo o mundo concluiu que a predominância do sexo masculino e a idade aumentaram com o tempo.

### Fatores de risco

Três condições subjacentes podem predispor o paciente a adquirir EI:

1. Doença valvular cardíaca e prótese valvular cardíaca, enxertos ou dispositivos
2. Cardiopatia congênita (CC)
3. História prévia de EI

A doença valvular cardíaca contribui fortemente para o volume de pacientes cardíacos nos centros de saúde, com uma prevalência importante na comunidade,^13^ resultante de uma maior expectativa de vida, envelhecimento populacional,^14^ e melhor atendimento médico e cirúrgico desses pacientes. Um declínio na doença valvar reumática foi observado nas últimas décadas,^14^ com a etiologia degenerativa como a causa mais comum em países desenvolvidos. No entanto, a carga da doença valvar reumática persiste em países de rendas baixa e média, com prevalência (a doença afeta até 7 em 1000 crianças escolares no Brasil, versus 0,1-0,4 em 1000 nos EUA)^18^ e mortalidade (275 mil mortes por ano no mundo) significativas.^17^ Um estudo recente de Glaser et al.^19^ indicou que as bioprósteses têm maior risco de infecção em comparação a válvulas mecânicas, mas mais estudos ainda são necessários.

Além disso, o implante de próteses, enxertos ou dispositivos cardíacos está aumentado continuamente, com impacto crescente sobre o número de infecções nesses implantes. Estima-se que 25-30% de todos os casos de EI ocorram em próteses valvares, de acordo com registros dos estudos Euro-Heart Survey de 2005,^20^ ICE de 2009,^4^ GAMES de 2015^5^ e EURO-ENDO de 2019.^6^ O implante de válvula aórtica transcateter (TAVI) tem sido cada vez mais utilizado em pacientes com estenose aórtica sintomática grave.^21^ Uma metanálise^22^ de quatro estudos, com um total de 3761 pacientes, publicada em 2019, concluiu que o risco de EI com TAVI não foi diferente quando comparado ao risco com cirurgia convencional de substituição da válvula aórtica.

As infecções relacionadas a marca-passo permanente e a cardioversor desfibrilhador implantável também têm aumentado ao longo do tempo,^23^ e correspondem a cerca de 10% dos episódios de EI.^4 , 5 , 24^

Em relação às CCs, a incidência acumulada em 25 anos^25^ de EI pós-cirurgia variou entre 1,3 e 13,3%, sendo mais elevada no grupo com estenose valvar aórtica. De fato, CC complexa, defeito do septo ventricular, válvula aórtica bicúspide, tetralogia de Fallot, e substituição da válvula aórtica constituem importantes fatores predisponentes para EI,^26^ com alto risco de mortalidade, cuja taxa foi estimada entre 6 e 14%.^26^

Séries de acompanhamento em longo prazo de pacientes com EI revelam que uma proporção significativa de pacientes que sobrevivem ao primeiro episódio de EI têm alto risco de recidiva (novo episódio de EI causada pelo mesmo microrganismo dentro dos primeiros seis meses após o episódio inicial^30^ ) ou reinfecção^31^ (infecção por um microrganismo diferente), estimado em 2,6-8,8%,^31 , 33^ com uma alta taxa de complicações e mortalidade.^34 , 36^

Outras condições importantes aumentam o risco de EI e necessitam ser consideradas clinicamente.

Apesar de o uso de drogas injetáveis, principalmente opioides, estar possivelmente diminuindo na União Europeia, o risco de infecções sanguíneas ainda é alto, com um aumento na infecção por *Staphylococcus aureus* sensível à meticilina e *Staphylococcus aureus* resistente à meticilina registrada nos últimos seis anos.^37^

A evidência crescente de bacteremia induzida por manipulação vascular e por cateter^38 , 39^ pode explicar o risco aumentado de EI no setor da saúde,^40 , 41^ atingindo até 35% das coortes, no setor de tatuagem e *body piercing,* ^42^ e pacientes com doença renal crônica em hemodiálise,^43 , 44^ o que tem influenciado fortemente o padrão mais contemporâneo de microrganismos predominantes nessa doença.

Além da insuficiência renal crônica, outras comorbidades aumentam o risco de EI, tais como diabetes mellitus,^45^ doença pulmonar crônica,^46^ doença hepática crônica,^46^ câncer,^47 , 48^ em particular câncer colorretal e urogenital, e doença periodontal.^49^

## Diagnóstico

### O papel do exame por imagem

Embora a história e o exame clínicos sejam fundamentais no diagnóstico de EI, o exame por imagem contribui exponencialmente para essa confirmação.

A ecocardiografia, pilar na prática clínica diária, desenvolveu consideravelmente, desde o exame bidimensional, ecocardiograma transesofágico (ETE),^50^ imagem harmônica,^51 , 52^ até a contribuição valiosa do ETE tridimensional na imagem de prótese valvar,^53^ melhorando a sensibilidade do ecocardiograma em detectar endocardite e complicações cardíacas locais.

No entanto, os critérios modificados de Duke ainda têm um papel limitado na confirmação do diagnóstico de casos mais complexos, tais como próteses valvulares, dispositivos cardíacos, e casos de hemoculturas negativas.^54^

Novas modalidades de imagem, tais como tomografia computadorizada (TC) cardíaca e TC por emissão de pósitrons com fluorodeoxiglicose (^18^ FDG-PET) ou cintilografia com leucócitos marcados (TC por emissão de fóton único - SPECT)^55^ parecem complementar a ecocardiografia, especialmente em próteses valvares,^56^ com melhora na sensibilidade quando associados aos critérios de Duke modificados. Por isso, em 2015, a Sociedade Europeia de Cardiologia (ESC, *European Society of Cardiology* )^57^ publicou novos critérios baseados nos critérios modificados de Duke incluindo o valor adicional (critérios “maiores”) dessas novas técnicas de imagem.

Ainda, a busca ativa por eventos embólicos ou aneurismas infecciosos por ressonância magnética (RM), TC de corpo inteiro, e/ou PET/CT foi adicionada como critério “menor”.

### Microbiologia

Praticamente qualquer agente pode causar EI, embora os mais frequentes sejam bactérias gram-positivas, principalmente *Staphylococcus* e *Streptococcus* , e mais recentemente, *Enterococcus* .

Na década de 70, estudos com pacientes internados revelaram *Streptococcus viridans* como o agente causal mais frequente de EI,^58^ e simultaneamente reconheceram que a frequência de infecção por *Staphylococcus* estava crescendo em pacientes com IE. Entre as *Streptococcus spp* , a mais frequente é *Streptococcus viridans* (um patógeno comum da mucosa oral), seguido por *Streptococcus bovis* (associado com neoplasias do cólon). Em 2007, uma metanálise^61^ concluiu que a incidência de *Staphylococci* e *Enterococci* aumentava, enquanto a incidência de EI causada por *Streptococci* e EI com cultura negativa reduziam significativamente. Essa tendência é preocupante, uma vez que esses agentes estão associados a uma alta taxa de mortalidade,^26 , 40 , 62^ sendo localmente destrutivos, com alta capacidade de causar embolia séptica.^63^

De fato, uma revisão sistemática recentemente publicada^64^ sobre agentes causadores de EI em 105 estudos concluiu que *Staphylococcus aureus* era o agente mais comum; *S. viridans* também estava entre os agentes mais comuns nos subgrupos de pacientes pediátricos, pacientes com CCs, e usuários de drogas injetáveis. No entanto, não podemos descartar a possibilidade de um viés de seleção, uma vez que a maioria dos estudos incluídos eram europeus e norte-americanos, com menor representação da Ásia, América do Sul, e África, onde o *S. viridans* é ainda um patógeno muito relevante e comum, apesar do menor número de estudos com foco nesse agente.

As bactérias do grupo HACEK ( *Haemophilus species, Aggregatibacter species, Cardiobacterium hominis, Eikenella corrodens,* e *Kingella kingae* ), normalmente presentes na orofaringe, são descritas como bactérias fastidiosas com uma baixa taxa em culturas, e responsável por menos que 5% das EIs.

Em relação à EI com cultura negativa, sua ocorrência é de cerca de 10 a 20% dos casos, segundo a maioria dos estudos populacionais ( Tabela 1 ), com exceção do estudo escocês de Shah et al.^65^ que relatou uma alta taxa de 58% em sua coorte. O uso prévio ou concomitante de antibiótico é uma etiologia comum.^66^ Além disso, diferenças em amostragem e testes,^67^ bem como infecção causada por microrganismos fastidiosos, intracelulares ou de difícil cultura também contribui para EI com cultura negativa. Tem se observado um atraso no diagnóstico clínico e escolha de um regime de antibióticos associados com deterioração hemodinâmica,^66 , 68^ embora haja evidências conflitantes sobre seu impacto sobre a mortalidade.^69^ Ainda, deve-se suspeitar de fungos e bactérias fastidiosas, e realizar culturas em meios específicos, considerando-se que uma baixa taxa de crescimento é esperada. Devem ser realizados testes sorológicos para Coxiella burnetii, Bartonella spp, Aspergillus spp., Mycoplasma pneumonia, Brucella spp. e Legionella pneumophila. Testes de reação em cadeia da polimerase (PCR) para Tropheryma whipplei, Bartonella spp e fungos (Candida spp, Aspergillus spp)^57^ podem ser realizados, apesar de sua baixa especificidade.^70^ Na área da cirurgia, Brandão et al.^71^ relataram que a inclusão da análise histopatológica e de PCR em válvulas cardíacas explantadas cirurgicamente foi mais útil no diagnóstico da causa da EI que somente a cultura da válvula.

Assim, uma abordagem sistemática, incluindo história completa do paciente (aspectos geográficos, viagem recente, contato com animais), histopatologia, investigações baseadas na cultura e aspectos moleculares e sorológicos é essencial na prática diária^64^ para aumentar a probabilidade de se identificar o agente causal.

### Manejo e desfechos

#### Antibióticos

A escolha dos antimicrobianos enquanto se aguarda resultado de culturas, ou quando o microrganismo responsável é conhecido, bem como na EI com cultura negativa, está bem definida e disponível nas diretrizes da ESC e da *American Heart Association* .^72^ A terapia é normalmente longa e por via parenteral.

O período de tratamento com antibióticos deve ser calculado a partir do primeiro dia do tratamento efetivo. Somente em caso de cultura da válvula cirúrgica positiva o tempo de terapia deve ser reiniciado, contando a partir da data da cirurgia; se não, a administração de antibióticos por mais duas semanas parece segura.^46 , 73^ A administração de antibióticos por um longo prazo é a regra, variando de duas a quatro semanas para *Streptococcus* oral em EI de válvula nativa, a seis semanas na infecção por *Enterococcus* ; EI de prótese valvar requer um período de tratamento de seis semanas. Isso significa um tempo de internação prolongado para completar o ciclo completo de antibióticos.

A terapia antimicrobiana parenteral ambulatorial ( *Outpatient Parenteral Antimicrobial Therapy* – OPAT) é geralmente usada para administração de terapia antimicrobiana parenteral em pelo menos duas doses em dias diferentes sem hospitalização,^74^ e tem sido usada em diferentes doenças infecciosas tais como pneumonia, pielonefrite, osteomielite, infecção de pele, diminuindo o tempo de internação. Em relação à EI, diretrizes europeias atuais apoiam o uso de OPAT em pacientes com endocardite após duas semanas de internação e em pacientes cooperativos e clinicamente estáveis (a OPAT pode ser iniciada mais cedo na infecção por *Streptococci* oral nativa ou *Streptococcus bovis* ) na presença de um programa ambulatorial bem estabelecido, com avaliação diária por um(a) enfermeiro(a) e semanalmente por um médico experiente.

Contudo, a terapia parenteral ambulatorial também tem limitações: a terapia parenteral prolongada pode ser desafiadora logisticamente, e difícil em caso de usuários de drogas intravenosas ou pacientes com câncer com acesso venoso deficiente. Há poucos estudos investigando o uso de antibióticos orais para completar o ciclo de antibiótico na EI.^75^ Após um curto período de terapia tripla de antibióticos intravenosa, ciprofloxacino e rifampicina orais mostraram-se efetivos em um pequeno ensaio com usuários de drogas endovenosas com EI do lado direito não complicada causada por *Staphylococcus* , em que a terapia parenteral não era viável.^76^ Um estudo recente, POET,^77^ também testou a eficácia e a segurança de se trocar a terapia intravenosa por oral em 400 pacientes estáveis com EI no lado esquerdo do coração. Os autores concluíram que a mudança para o tratamento oral não foi inferior ao regime parenteral contínuo convencional nesses pacientes.

#### Cirurgia

A cirurgia tem um papel crucial na EI.^78^ A Europa apresenta taxas mais altas de intervenções cirúrgicas na EI em comparação ao restante do mundo. Séries populacionais apresentam taxas entre 15% e 50% ( Tabela 1 ). Nos registros do EURO-ENDO^6^ ou ICE,^4^ quase metade dos pacientes foram operados.

Vários estudos observacionais concluíram que a cirurgia tem efeito protetor durante a fase ativa da EI.^80^ Entretanto, nem todos os pacientes com indicação clínica para cirurgia são de fato operados. O registro ICE-PLUS^84^ e o estudo GAMES^5^ estimaram que aproximadamente um quarto dos pacientes com indicação cirúrgica não foram submetidos à cirurgia. Razões para isso incluíram um prognóstico ruim, instabilidade hemodinâmica, acidente vascular cerebral, sepse, e morte antes da cirurgia. Ainda, observou-se uma concordância moderada entre a prática clínica e as diretrizes recomendadas em relação às indicações cirúrgicas na EI.^85^

O melhor momento para se realizar a cirurgia ainda é um ponto controverso; o “precoce” e o “tardio” podem ter diferentes traduções. Enquanto diretrizes europeias^57^ enfatizam a que a cirurgia deve ser realizada em caráter de emergência (dentro de 24 horas) ou urgência (dentro de poucos dias), diretrizes americanas^42^ referem-se à cirurgia “precoce” como aquela realizada durante internação inicial e antes de se completar um ciclo completo de antibióticos. Estudos observacionais mostraram uma redução na mortalidade hospitalar com cirurgia precoce,^83 , 86 , 87^ e uma meta-análise conduzida em 2016 também demonstrou um papel protetor da cirurgia precoce sobre o prognóstico.

Em relação ao tipo de procedimento na válvula, deve-se fazer uma escolha entre reparo e substituição da válvula. Diretrizes internacionais^88^ enfatizam que o reparo da válvula deve ser a opção de escolha em válvulas nativas com envolvimento limitado dos folhetos ou cúspides. Em um estudo populacional^89^ referente ao estado de Nova Iorque e ao estado da Califórnia nos EUA, 19% dos pacientes com EI de válvula mitral nativa foram submetidos a reparo da válvula, o que foi associado a uma melhor taxa de sobrevida e menor risco de recorrência. No entanto, tal fato pode não representar a prática na vida real. Por outro lado, se a destruição da válvula nativa for grave, a escolha pelo tipo de prótese deve considerar fatores relacionados ao paciente, tais como idade, adesão a anticoagulantes, e expectativa de vida. De fato, atualmente, não existe evidência sobre a superioridade das biopróteses ou das válvulas mecânicas,^90^ uma vez que elas apresentam taxas similares de sobrevida e recorrência de endocardite.^91^

Além disso, vários grupos têm conduzido uma busca contínua por um escore prognóstico ideal para estratificação de risco na cirurgia cardíaca na EI,^92^ embora nenhum escore de risco atualmente tem se mostrado superior aos demais na EI.^96^ A estratificação de risco antes da cirurgia é, no entanto, crucial, e deve levar em consideração o estado clínico, comorbidades, e risco cirúrgico do paciente.^57^ Há um papel decisivo da equipe multidisciplinar na abordagem da EI, no encaminhamento do paciente, em tempo hábil, para avaliação cirúrgica e clínica, especialmente em casos de EI do lado esquerdo.^57 , 69^

A mortalidade pós-cirúrgica varia entre 6 e 29% em estudos observacionais ( Tabela 2 ). Uma metanálise publicada em 2019 por Varela-Barca^79^ identificou os seguintes fatores associados à maior mortalidade após cirurgia: idade, sexo feminino, cirurgia de urgência ou emergência, cirurgia cardíaca prévia, classe NYHA ≥ III, choque cardiogênico, prótese valvar, envolvimento de múltiplas válvulas, insuficiência renal, abscesso perivalvular e infecção por *Staphylococcus aureus* .

**Tabela 2:** – Características de séries cirúrgicas observacionais sobre pacientes com endocardite infecciosa obtidas

Autores/estudo	País	Delineamento do estudo	Período de tempo	N	Válvula nativa (%)/Prótese valvar	Taxa de mortalidade (%)
Jassal et al.^115^	EUA	Retrospectivo	1995-2004	91	85,7/14,3	15,4
Bannay et al.^116^	França	Prospectivo	1998-2000	240	67,5/32,5	19,4
Lalani et al.^83^	Multicêntrico	Prospectivo	2000-2005	720	100/0	12,1
Gaca et al.^95^	EUA	Retrospectivo	2002-2008	19543	ND	8,2
Lalani et al.^86^	Multicêntrico	Prospectivo	2000-2005	490	0/100	22
Pang et al.^117^	Singapura	Retrospectivo	2000-2012	191	92,7/7,3	6,3
Machado et al.^118^	Brasil	Prospectivo	2003-2010	64	ND	17
Madeira et al.^94^	Portugal	Retrospectivo	2007-2014	128	75,7/15	16
Olmos et al.^97^	Espanha	Prospectivo	1996-2014	671	60/40	28,6
Pivatto et al.^93^	Brasil	Retrospectivo	2007-2016	107	-/31	29
Varela et al.^119^	Espanha	Retrospectivo	2002-2016	180	62,6/37,5	26,8
Guiomar et al.^120^	Portugal	Retrospectivo	2006-2017	145	68/32	13,1

*ICE-PCS: International Collaboration on Endocarditis–Prospective Cohort Study; ND: não disponível.*

Embora as taxas de cirurgia tenderam a aumentar em 7% por década de 1969 a 2000,^61^ desde o início deste século, a tendência geral é em direção a estabilidade,^4 , 8 , 9 , 61^ mesmo estudos populacionais conduzidos na Espanha^97^ e nos EUA^98^ terem apresentado taxas crescentes continuamente. Isso provavelmente é resultante das diretrizes científicas recentes, dos avanços contínuos no cuidado intensivo e manejo cirúrgico desses pacientes.

#### Mortalidade

A taxa de mortalidade hospitalar varia entre países, de 8 a 40%.^12^ Em relação à mortalidade em curto prazo (até 30 dias de acompanhamento) relatada em estudos populacionais nas duas últimas décadas, as taxas variam entre 11e 25%, enquanto um seguimento de um ano revelou uma taxa de 32% ( Tabela 1 ). Uma metanálise publicada em 2017^99^ incluindo 25 estudos observacionais, estimou uma taxa de mortalidade de curto (seis meses) e longo prazo (até 10 anos) de 20% e 37%, respectivamente. Fernandez-Hildago et al.,^33^ Toyoda et al.^11^ e Ilhão Moreira et al.^80^ relataram uma taxa de mortalidade de cinco anos de 52%, 53% e 43%, e Netzer et al.^100^ relataram uma taxa de mortalidade em sete anos de 56%.^100^ Apesar da escassez dos de acompanhamento de longo prazo, os dados disponíveis revelam uma tendência de um prognóstico ruim desses pacientes, mesmo se sobrevivem à hospitalização por EI ativa.

Vários fatores têm sido associados à mortalidade relacionada ao aumento da EI. Em 2019, uma meta-análise^79^ com 16 estudos, incluindo 7484 pacientes, identificou que sexo feminino, cirurgia de urgência ou emergência, cirurgia cardíaca prévia, classe NYHA ≥ III, choque cardiogênico, prótese valvar, comprometimento de múltiplas válvulas, insuficiência renal, abscesso perivalvular e infecção por *Staphylococcus aureus* como marcadores importantes de mortalidade hospitalar.

As causas de morte têm sido pouco abordadas na maioria das séries. Fernandez-Hildago et al.^33^ descreveram, em estudo observacional prospectivo do tipo coorte com 438 pacientes com EI, uma taxa de mortalidade hospitalar de 29%, 80% das mortes diretamente relacionadas à EI, enquanto as mortes restantes deveram-se, principalmente, à infecção nosocomial ou hemorragia importante. Registros prospectivos^6 , 20^ identificaram causas cardiovasculares, principalmente insuficiência cardíaca, e sepse, como as principais causas de mortalidade hospitalar nesses pacientes. Causas de mortalidade em longo prazo não foram exploradas.

#### Profilaxia da EI

Em 2007 nos EUA,^101^ e em 2009 na Europa,^57^ as indicações para profilaxia antibiótica foram reduzidas, com importantes limitações quanto ao uso de antibióticos durante procedimentos dentários, e interrupção do tratamento durante procedimentos geniturinários e gastrointestinais. Em 2008, o Instituto Nacional de Excelência em Saúde e Assistência do Reino Unido^102^ emitiu diretrizes abolindo todas as indicações para o uso de profilaxia antibiótica para procedimentos dentários e não dentários.

De acordo com diretrizes europeias,^57^ a profilaxia antibiótica deve ser administrada para pacientes de alto risco:

1. Pacientes com protese valvar ou com material de prótese usado para reparo de válvula cardíaca;
2. Pacientes com EI prévia;
3. Pacientes com CC cianótica não tratada, e pacientes com CC com derivações e condutos paliativos, ou outras próteses.

Neste subgrupo de pacientes, a profilaxia antibiótica deve ser usada para procedimentos dentários que requeiram manipulação da região gengival ou periférica dos dentes, ou perfuração da mucosa oral.

Porém, tais decisões não são consensuais entre os países. Países da América Latina, incluindo o Brasil, permanecem conservadores. A profilaxia antibiótica na EI ainda inclui pacientes com doença valvar significativa, tais como válvula aórtica degenerativa ou bicúspide, prolapso da válvula mitral com regurgitação ou doença valvar reumática. Ainda, a profilaxia é usada antes de procedimentos geniturinários ou gastrointestinais que envolvam mucosa em pacientes de alto risco.^103^

## Tendências da incidência e da mortalidade

Em países desenvolvidos como Dinamarca, Itália, Inglaterra, Espanha, Alemanha, Finlândia e Países Baixos, tem se observado uma tendência de aumento da incidência de EI nas duas últimas décadas ( Tabela 3 ). Tal fato pode ser explicado por razões demográficas (por exemplo, envelhecimento populacional), mudanças na etiologia da valvulopatia, número crescente de pacientes com implante de próteses ou dispositivos cardíacos, aumento na sobrevida de pacientes com doença cardíaca estrutural ou congênita, necessidade de uso prolongado de acesso vascular em diferentes condições clínicas, e avanços nas medidas profiláticas.

**Tabela 3:** – Evolução das taxas de incidência e mortalidade nos estudos populacionais sobre endocardite infecciosa

Autores/estudo	País	Período de tempo	Tendência de incidência crescente	Tendência das taxas de cirurgia	Tendência da mortalidade
Fonager et al.^1^	Dinamarca	1980-1997	Sim	ND	Decrescente
Tleyjeh et al.^2^	EUA (Condado de Olmsted)	1970-2000	Não	Estável	Estável
Sy et al.^7^	Austrália (Nova Gales do Sul)	2000-2006	Não	ND	Estável
Fedeli et al.^8^	Itália (Veneto)	2000-2008	Sim	Estável	Crescente
Bikdeli et al.^109^	EUA	1999-2010	Não	ND	Estável
Duval et al.^9^	França (grande Paris, Lorraine e Ródano-Alpes)	Inquérito de um ano (1991, 1999 e 2008)	Não	Aumento de 1991 a 1999. Em seguida, estável.	ND
Dayer et al.^111^	Inglaterra	2000-2013	Sim (mais pronunciada após 2008)	ND	ND
Pant et al.^112^	EUA	2000-2011	Sim (para EI por *Streptococcus* após 2007)	Aumento 2000-2007/estável 2007-2011	ND
Erichsen et al.^10^	Dinamarca	1994–2011	Sim (em homens e idosos)	ND	ND
Keller et al.^62^	Alemanha	2005-2014	Sim (mais pronunciado após 2009)	ND	Estável
Toyoda et al.^11^	EUA (Califórnia e estado de Nova Iorque)	1998-2013	Não	ND	Estável
Van den Brink et al.^113^	Países Baixos	2005-2011	Sim	ND	ND
Cresti et al.^3^	Itália (Grosseto)	1998-2014	Sim	ND	Aumento leve (p=0,055)
Olmos et al.^97^	Espanha	2003-2014	Sim	Aumento	Decrescente (0,2%/ano)
Ahtela et al.^114^	Finlândia	2005-2014	Sim	ND	Estável
Shah et al.^65^	Escócia	1990-2014	Não (aumento em pacientes >80 anos de idade)	ND	ND
Khan et al.^98^	EUA	2002-2016	ND	Aumento	Decrescente (de 16,7% a 9,7%)

*ND: não disponível.*

Por outro lado, essa tendência não é observada em outros países como França, Austrália, Escócia ou Estados Unidos.^2 , 9^ Essas discrepâncias estão provavelmente relacionadas a diferentes fontes e definição de casos, e timing do impacto da melhoria nos métodos diagnósticos (imagem, microbiologia).

O maior impacto da profilaxia da EI sobre a incidência da EI em pacientes de alto risco (diretrizes da American Heart Association de 2007^101^ e da Sociedade Europeia de Cardiologia em 2009^104^ ) foi avaliado em diferentes estudos, mas incertezas persistem, conforme mostrou uma metanálise de Khan et al.^105^ publicada em 2016. De fato, enquanto alguns autores mostraram um aumento mais evidente na incidência de EI em países como Reino Unido, Alemanha e Holanda ( Tabela 3 ), DeSimone et al.^106^ e Duval et al.,^9^ dos Estados Unidos e França, respectivamente, não observaram essa tendência. Assim, esforços são necessários para a avaliação do impacto de diretrizes locais e adesão por parte dos médicos sobre a incidência da EI no mundo.

Apesar dos avanços significativos no diagnóstico e no manejo (clínico e cirúrgico) da EI, observa-se estabilidade em relação à mortalidade na maioria das séries populacionais. Exceções foram Itália, onde se observou um aumento,^3 , 8^ e Dinamarca,^1^ Espanha,^97^ e Estados Unidos,^98^ onde registrou-se diminuição da mortalidade. Os registros ICE^4^ e EURO-ENDO^6^ apresentaram uma taxa de mortalidade moderadamente maior de 18% e 17%, respectivamente, em comparação ao Euro Heart Survey (13%).^20^ Finalmente, em 2013, uma meta-análise^12^ mostrou uma diminuição na mortalidade hospitalar de 1960 a 1980, com estabilidade desde então.

## Desafios e direcionamentos futuros

No último século, avanços cirúrgicos e clínicos permitiram uma melhora marcante no manejo e prognóstico da EI. Porém, médicos ainda enfrentam desafios diários no manejo desses pacientes ( Tabela 4 ).

**Tabela 4:** – Principais desafios atuais da endocardite infecciosa

Área principal	Desafios
Prevenção	- profilaxia da EI (uso de indicações mais restritas ou mais amplas) - conhecimento por parte de médicos e pacientes
Diagnóstico	- critérios - culturas de sangue negativas - dispositivos e próteses cardíacas – valor do teste de imagem
Terapia clínica	- evidência robusta (ensaios clínicos randomizados controlados) - uso de terapia antimicrobiana parenteral ambulatorial
Cirurgia	- evidência robusta (ensaios clínicos randomizados controlados) - timing - reparo *versus* substituição de válvula (expertise técnica) - em pacientes com acidente vascular cerebral isquêmico - em usuários de drogas intravenosas - na endocardite de próteses (“sempre” versus “ocasionalmente”)
Manejo	- equipe de cardiologia *versus* equipe multidisciplinar - centros de referência

*EI: endocardite infecciosa.*

A prevenção deveria ser uma prioridade nas políticas de saúde nacionais. Campanhas educacionais a médicos e pacientes são de suma importância e a profilaxia da EI, analisando-se quais pacientes mais se beneficiariam, deve ser otimizada.

Devem ser criados centros especializados reunindo experts em diagnóstico da EI por imagem, doença infecciosa, e cardiologia, objetivando-se melhores desfechos clínicos e cirúrgicos. Ainda, deve-se estimular a comunicação direta com outros centros. Protocolos de imagem multimodais devem ser estabelecidos, bem como pesquisas sobre melhorias tecnológicas devem ser realizadas. A necessidade de se reduzir o tempo de internação hospitalar, com o estabelecimento de equipes ambulatoriais bem treinadas e pacientes devidamente orientados, o que permitiria a realização de OPAT sempre que possível, também deve ser estimulado pelas instituições.

Estudos baseados em evidência são ainda bem excepcionais e globalmente heterogêneos. De fato, a maioria dos nossos dados foram obtidos de registros, estudos populacionais, e experiências unicêntricas e multicêntricas de país de renda média e alta, o que leva a um possível viés de seleção ao considerar a realidade mundial. Ensaios controlados randomizados devem ser realizados para fornecer mais evidências acerca do momento adequado para cirurgia, esquemas de antibióticos, o efeito da terapia clínica adjuvante durante tratamento ativo da IE e uso de material prostético menos vulnerável à adesão por bactérias.

Um comentário final sobre o uso de redes de inteligência artificial atualmente desenvolvidas em centros de grande porte,^107^ que permitirá uma estimativa precisa do risco de complicações e do momento ideal para cirurgia, levando a um melhor prognóstico do paciente.
