# Supplementary material for: Endocardite Infecciosa: Ainda mais Desafios que Certezas
Source: Arq Bras Cardiol. 2022 May 4;118(5):976–88. [Article in Portuguese] doi: 10.36660/abc.20200798 (PMC9368884; doi:10.36660/abc.20200798)
Supplement: Translation

# Translation: Infective Endocarditis: Still More Challenges Than Convictions

**Authors:** Catarina Sousa, Fausto J. Pinto

**Affiliations:** 1 Portugal Centro Cardiovascular Universidade de Lisboa (CCUL), Faculdade de Medicina, Universidade de Lisboa, Lisboa – Portugal; 2 Portugal Serviço de Cardiologia, Centro Hospitalar Barreiro Montijo (CHBM), Barreiro – Portugal; 3 Portugal Departamento Coração e Vasos, Centro Hospitalar e Universitário Lisboa Norte (CHULN), Lisboa – Portugal

**Keywords:** Infective Endocarditis, Epidemiology, Diagnosis, Prophylaxis

## Abstract

After fourteen decades of medical and technological evolution, infective endocarditis continues to challenge physicians in its daily diagnosis and management. Its increasing incidence, demographic shifts (affecting older patients), microbiology with higher rates of *Staphylococcus* infection, still frequent serious complications and substantial mortality make endocarditis a very complex disease. Despite this, innovations in the diagnosis, involving microbiology and imaging, and improvements in intensive care and cardiac surgical techniques, materials and timing can impact the prognosis of this disease. Ongoing challenges persist, including rethinking prophylaxis, improving the diagnosis criteria comprising blood culture-negative endocarditis and prosthetic valve endocarditis, timing of surgical intervention, and whether to perform surgery in the presence of ischemic stroke or in intravenous drug users. A combined strategy on infective endocarditis is crucial, involving advanced clinical decisions and protocols, a multidisciplinary approach, national healthcare organization and health policies to achieve better results for our patients.

“ *It is of use from time to time to take stock, so to*

*speak, of our knowledge of a particular disease, to see*

*exactly where we stand in regard to it, to inquire to what*

*conclusions the accumulated facts seem to point, and*

*to ascertain in what direction we may look for fruitful*

*investigations in the future* .”

*William Osler (1885)*

## Epidemiology

### Incidence and demographics

The incidence of infective endocarditis (IE) varies between 3 and 15 cases per 100,000 in population-based studies^1^ ( Table 1 ). This variation is probably related to several factors: case definition criteria (definitive case, possible case, inclusion of blood culture-negative IE), different sources of cases or the time period analyzed with reference to the publication of the guidelines. In this analysis, we did not include single-center or multicentric observational studies with a high risk of selection bias that could therefore underestimate the real incidence of this disease.

**Table 1:** – Population studies on infective endocarditis incidence, demographic and outcome features

Authors/study	Country	Population Size (Source)	Time period	Source list of cases	Number of cases	Crude incidence/100,000	Age range	Male/Female ratio	Negative Blood culture (%)	Outcomes: Valve surgery and mortality
Fonager et al.^1^	Denmark	5.2 million	1980-1997	Danish National Registry	3,351	Gender analysis: Men 4 - 6 Women 3 - 4	60.4	1.32	NA	30-d case fatality rate 23%
Tleyjeh et al.^2^	USA (Olmsted County)	1970 - 51 000 2000 - 90 000	1970-2000	Rochester Epidemiology Project	107	5.0-7.0	61.5 (18.8-90.6)	2.7	1	Valve surgery 15% 6 mo mortality 14-33%
Sy et al.^7^	Australia (New South Wales)	6.6 million	2000-2006	Statewide database	1,536	4.7	62 (42-75)	1.8	15	Valve surgery 20% In Hospital mortality 14% 6 mo. mortality 18%
Fedeli et al.^8^	Italy (Veneto region)	4.8 million	2000-2008	Electronic archives of hospital discharge records	1,863	4.4	68	1.4 - 2	NA	Valve surgery 23% In Hospital mortality 14.3%
Selton-Suty et al.^108^	France (greater Paris, Lorraine, Rhone-Alpes, Franche-Comte´, Marne, Ille-et-Vilaine, and Languedoc-Roussillon)	15.3 million	2008	Case report forms sent to physicians likely to be in charge of IE cases	497	3.2	62.3	2.8	5	Valve surgery 44.9% In-hospital mortality 22.7%
Bikdeli et al.^109^	USA	NA (inpatients >65 yrs. old)	1999-2010	Medicare & Medicaid Services, Medicare inpatient Standard Analytic Files	262,658	7.2	79	0.7-0.8	NA	In hospital mortality 9-11% 30 d mortality – 14-16.5% 1 yr. mortality – 32.6-36.2%
Duval et al.^9^	France (Greater Paris, Lorraine, and Rhône-Alpes)	11 million	1 yr. survey 1991, 1999 and 2008	Case report forms sent to physicians likely to be in charge of IE cases	1991 - 323 1999 – 331 2008 - 339	1991 – 3.5 1999 – 3.3 2008 – 3.2	1991 – 57.9 1999 – 59.8 2008 – 61.6	1991 – 1.9 1999 – 2.3 2008 – 2.9	NA	Valve surgery (1991 – 31.3%; 1999 – 50.2%; 2008 – 49.6%) In Hospital mortality (1991 – 20.7%; 1999 – 15.4%; 2008 – 21.2%)
Ternhag et al.^110^	Sweden		1997-2007	Swedish Hospital Discharge Register	7,603	7.7	65.7	1.45	NA	30-day mortality rate – 10.4% 1-5 yr. mortality rate – 14.7%
Dayer et al.^111^	England	NA	2000-2013	Secondary Uses Service	19,804	2.7	NA	NA	NA	NA
Pant et al.^112^	USA	NA	2000-2011	Nationwide Inpatient Sample database	457,052	11-15	NA	NA	NA	NA
Erichsen et al.^10^	Denmark	NA	1994–2011	Danish nationwide registry	5,486	7.55 (2009–2011)	63	1.8	NA	NA
Keller et al.^62^	Germany	80 million	2005-2014	Nationwide database of the Federal Statistical Office of Germany	94,364	11.6	NA	NA	NA	In hospital mortality 17%
Toyoda et al.^11^	USA (California and New York State)	NA	1998-2013	Statewide Planning and Research Cooperative System database (New York); Office of Statewide Health Planning and Development database (California)	75,829	7.6 to 9.3	62.3	1.2	25	Valve surgery - 11-13% 90-day mortality – 24%
Van der Brink et al.^113^	Netherlands	NA	2005-2011	Dutch Healthcare Authority	5213	2005-3.0 2011- 6.3	66.4	2.3	9.3	All-cause mortality 36.1%
Cresti et al.^3^	Italy (Grosseto)	217778	1998-2014	Healthcare system database	170	4.6	69.5	1.54	19	Valve surgery – 46% In hospital mortality 25% 1 yr. mortality 32%
Ahtela et al.^114^	Finland	NA	2005-2014	Care Register for Health Care (CRHC) data- base	2611	6.3	60.0	2.1	NA	30 d mortality – 11%
Olmos et al.^97^	Spain	NA	2003-2014	Spanish National Health System	16867	2.7 in 2003 3.5 in 2014 per 100,000 person/year	NA	2	NA	In hospital mortality rate 20%
Shah et al.^65^	Scotland	NA	1990-2014	National hospitalization registry National microbiology register	7638	5.3 in 1990	65	0.96	58	30-day outcome: All cause death – 9.6-17.3% Valve surgery – 3.8-6.3% 1-year outcome: All cause death – 27-33.3% Valve surgery – 9.2-15.5%

*NA: non-available; IE: infective endocarditis.*

A male predominance can be noted with a male: female ratio varying between 0.96 and 2.8. This is also observed in large international registries such as the ICE study,^4^ the GAMES registry in Spain^5^ or the recently published EURO-ENDO.^6^

In most populational series and international registries, older patients are normally more affected, the median age from late 50s to 60s ( Table 1 ). Also, the incidence increases with aging.^7 - 11^

Finally, a meta-analysis published in 2013^12^ that included 160 studies worldwide concluded that male gender predominance and age increased over time.

### Risk factors

Three main underlying conditions usually predispose patients to acquire IE:

1. Heart valve disease and cardiac valve prothesis, grafts or devices
2. Congenital heart disease (CHD)
3. Previous history of IE

Heart valve disease is a major contributor to the pool of cardiac patients in daily clinical setting, with a significant prevalence in the community,^13^ as a result of higher life expectancy, aging of the populations^14^ and improved medical and surgical care of valvular patients. A decline in rheumatic valve disease was noted in the last decades,^14 - 17^ with degenerative etiology being the most prevalent in developed countries. Nevertheless, the burden of rheumatic valve disease persists in low-to-middle income countries with significant prevalence (in Brazil, it affects up to 7/1000 school children versus 0.1-0.4/1000 in the USA)^18^ and mortality (275,000 deaths each year worldwide).^17^ A recent study by Glaser et al.^19^ indicated that bioprostheses have a higher risk of infection compared to mechanical valves, but more studies are still needed.

Also, the implantation of cardiac prothesis, grafts or devices is continually increasing, with a growing impact on the number of infections in these implants. It has been estimated that 25-30% of all cases of IE occur in prosthetic valves, according to the registries of the Euro-Heart Survey in 2005,^20^ ICE in 2009,^4^ GAMES in 2015^5^ and EURO-ENDO in 2019.^6^ Transcatheter aortic valve implantation (TAVI) has been increasingly used in severe symptomatic aortic stenosis.^21^ A metanalysis^22^ of four studies, with 3,761 patients, published in 2019, concluded that the risk of IE with TAVI was not different as compared with conventional surgical aortic valve replacement.

Infections related to permanent pacemakers and implantable cardioverter-defibrillators have also been increasing over time^23^ and account for about 10% of IE episodes.^4 , 5 , 24^

Regarding CHD, the 25-year cumulative incidence^25^ of IE after surgery varied between 1.3 and 13.3%, being highest in the aortic valve stenosis group. In fact, complex CHD, ventricular septal defect, bicuspid aortic valve, tetralogy of Fallot and aortic valve replacement, constitute important predisposing factors for IE^26 - 28^ and with a high mortality risk, estimated between 6 and 14%.^26 - 29^

Long-term follow-up series of IE patients reveal that a significant proportion of patients that survive their first episode of IE carry a higher risk of relapse (new IE episode caused by the same microorganism within the first six months after the initial episode^30^ ) or re-infection^31 - 33^ (infection by a different microorganism), estimated in 2.6-8.8%,^31 , 33 - 35^ with a high rate of complications and mortality.^34 , 36^

Other important conditions increase the risk of IE and need to be considered clinically.

Although the use of injection drugs, mainly opioids, may be decreasing in the European Union, the risk of blood infections remain high, with an increase in methicillin-sensitive and methicillin-resistant *Staphylococcus aureus* infection registered in the last six years.^37^

The growing evidence on vascular manipulation- and catheter-induced bacteremia^38 , 39^ can explain the increased risk of IE in the health care setting^40 , 41^ ranging up to 35% of total cohorts, in tattooing and body piercing^42^ and in patients with chronic renal disease on hemodialysis,^43 , 44^ which has strongly influenced the most contemporary pattern of predominant microorganisms in this disease.

Besides chronic renal failure, other comorbidities increase the risk of IE such as diabetes mellitus,^45^ chronic lung disease,^46^ chronic liver disease,^46^ cancer,^47 , 48^ in particular colorectal and urogenital cancer, and periodontal disease.^49^

## Diagnosis

### The role of imaging

Clinical history and examination are pivotal in the diagnosis of IE. Even so, imaging contributes exponentially for its confirmation.

Echocardiography, keystone in every day clinical practice, has developed considerably, from 2D, transesophageal echo (TEE),^50^ harmonic imaging,^51 , 52^ to the increment value of 3D TEE in prosthetic valve imaging,^53^ improving echocardiographic sensitivity to detect endocarditis and its local cardiac complications.

Nevertheless, the modified Duke criteria continue to have a limited role in confirming the diagnosis in more complex cases such as in prosthetic valves, cardiac device and Negative Blood Culture (NBC) cases.^54^

New imaging modalities, such as cardiac computed tomography (CT) and metabolic imaging by 18-fluorodeoxyglucose positron emission tomography (^18^ FDG-PET) or leukocyte scintigraphy (radiolabeled leukocyte single-photon emission CT [SPECT])^55^ have been shown to complement the use of echocardiography specially in prosthetic valves^56^ with improvement in sensitivity when aggregated to the modified Duke criteria. This fact led the European Society of Cardiology^57^ to issue, in 2015, a new set of criteria based on the modified Duke criteria with added value (major criteria) of these new imaging techniques.

Also, the active search of embolic events or infectious aneurisms by cerebral magnetic resonance imaging (MRI), whole-body CT and/or PET/CT was added as a minor criterion.

### Microbiology

Almost any agent can cause IE, although the most frequent are gram positive bacteria, namely *Staphylococcus* and *Streptococcus* and more recently *Enterococcus* .

In the 1970’s, hospital series reported *Streptococcus viridans* as the most frequent causal agent of IE,^58 - 60^ but simultaneously acknowledged that *Staphylococcus* frequency among IE patients was increasing. Among *Streptococcus spp* , the most frequent is *Streptococcus viridans* (a common pathogen in the oral mucosa) followed by *Streptococcus bovis* (associated with colonic neoplasms). In 2007, a metanalysis^61^ concluded on an increase incidence of *Staphylococci* and *Enterococci* with a significant decrease in IE caused by *Streptococci* and NBC IE. This trend is worrying as these agents are associated with a high mortality rate,^26 , 40 , 62^ being locally destructive with a high capacity to embolize (septic metastasis).^63^

In fact, a recently published systematic review^64^ concerning the causative agent of IE in 105 studies concluded that *Staphylococcus aureus* was the most common agent; *S. viridans* was also among the most common agents in the subgroups of pediatric and CHD patients, and intravenous drug users. A selection bias cannot be excluded, though, as most included studies were from Europe and North America, with less representation from Asia, South America and Africa, where *S. viridans* is still a very relevant and common pathogen, despite fewer studies focused on it.

The HACEK (H *aemophilus* species, *Aggregatibacter* species, *Cardiobacterium hominis, Eikenella corrodens* , and *Kingella kingae* ) microorganisms, normally present in the oropharynx, are described as fastidious bacteria with a low growth rate in cultures, and responsible for less than 5% of IE.

Regarding NBC IE, it occurs in about 10-20% of cases, according to most populational studies ( Table 1 ), excluding the Scotland study by Shah et al.^65^ that reported an unusually high rate of 58% in their cohort. Previous/concomitant antibiotic use is a common etiology.^66^ Sampling and testing differences,^67^ as well as infection due to fastidious, intracellular or challenging to culture organisms also contributes to blood culture-negative IE. A delay in the clinical diagnosis and choice of antibiotic regimen associated with hemodynamic deterioration has been observed,^66 , 68^ although conflicting evidence exists regarding its impact on mortality.^69^ Still, fungi and fastidious bacteria should be suspected and cultures in specialized media should be performed, considering that a slow growth rate is expected. Serological testing for Coxiella burnetii, Bartonella spp, Aspergillus spp., Mycoplasma pneumonia, Brucella spp. and Legionella pneumophila should be performed. Blood polymerase chain reaction (PCR) assays for Tropheryma whipplei, Bartonella spp and fungi (Candida spp, Aspergillus spp)^57^ can be performed although low sensitivity is acknowledged.^70^ In the surgical field, Brandão et al.^71^ reported that the inclusion of histopathologic and PCR analysis in surgically explanted cardiac valves proved more useful in diagnosing the IE etiology than valve culture by itself.

Therefore, a systematic approach with a complete patient’s history (including geography, recent travel, contact with animals), histopathology, culture-based, molecular and serological investigations are essential in every-day pratice^64^ to increase the likelihood of identifying the causal agent.

### Management and outcomes

#### Antibiotics

The selection of antimicrobials, either while waiting for the cultures, or when the responsible microorganism is known, and in NBC IE, is well defined and can be found in the European Society of Cardiogy^65^ and American Heart Association^72^ guidelines. Therapy is usually prolonged and parenteral.

The length of antibiotic therapy should be calculated from the first day the effective treatment was established. Only in case of a positive surgical valve culture should the time of antibiotic therapy be restarted, counting from the surgical date; otherwise it may be safe to administer antibiotic therapy for another two weeks.^46 , 73^ Long-term antibiotic administration is the rule, from two to four weeks in oral *Streptococcus* native-valve IE to six weeks in *Enterococcus* infection; prosthetic valve IE requires a six-week duration course. This usually means a prolonged hospital stay to complete the full cycle of antibiotic.

The Outpatient Parenteral Antimicrobial Therapy (OPAT) is generally used for delivery of parenteral antimicrobial therapy in at least two doses on different days without intervening hospitalization,^74^ and it has been used in different infectious settings such as pneumonia, pyelonephritis, osteomyelitis, skin infection, decreasing hospital length of stay. Regarding IE, current European guidelines support the use of OPAT in endocarditis patients after the first two weeks of hospitalization and in cooperative and medically stable patients (the OPAT can actually be started earlier in native valve oral *Streptococci* or *Streptococcus bovis* ) as long as an outpatient program is set with daily evaluation by a nurse and weekly by an experienced physician.

Nevertheless, parenteral outpatient therapy has also limitations: prolonged parenteral therapy can be logistically challenging and difficult in intravenous drug users or cancer patients with poor venous access. Few studies on the use of oral antibiotic to complete the full cycle of antibiotic therapy in IE have been performed.^75^ After a short course of triple intravenous antibiotic, oral ciprofloxacin and rifampicin has been shown to be effective in a small trial of uncomplicated right-side *Staphylococcus* IE in intravenous drug users where parenteral therapy was not feasible.^76^ A recent trial, POET,^77^ also tested the efficacy and safety of switching from intravenous to oral antibiotics in 400 stable patients who had left-sided IE. It concluded that changing to oral regimen was not inferior to continuous conventional parenteral regimen in these patients.

#### Surgery

Surgery plays a crucial role in IE.^78^ Europe presents higher rates of surgical intervention in IE than the rest of the world. Populational series present rates between 15% and 50% ( Table 1 ). At the EURO-ENDO^6^ or ICE^4^ registries, almost half the patients were operated.

Several observational studies have concluded on the protective effect of surgery during the active phase of IE.^80 - 83^ Nevertheless, not all patients with a clinical indication for surgery are in fact operated. The ICE-Plus registry^84^ and the GAMES study^5^ estimated that approximately a quarter of patients with surgical indication did not undergo surgery. Reasons for this included poor prognosis, hemodynamic instability, stroke, sepsis, and death before surgery. Also, only a moderate agreement was found between clinical practice and recommended guidelines regarding surgical indication in EI.^85^

The best timing for surgery continues to be controversial; “early” *versus* “late” may have different translations. While European guidelines^57^ emphasize that surgery should be performed on an emergent (within 24 h) or urgent (within a few days) basis, American guidelines^42^ refer to “early” surgery as during initial hospitalization and before completion of a full course of antibiotics. Observational studies have shown a reduction of in-hospital mortality with early surgery,^83 , 86 , 87^ and a metanalysis conducted in 2016 also concluded on the protective role of early surgery on prognosis.

Regarding the type of valve procedure, a choice between repair and replacement must be made. International guidelines^88^ emphasize that valve repair should be the option in native valves with limited involvement of leaflets or cusps. In a population-based study^89^ concerning the New York State and California, USA, 19% of patients with native mitral valve IE underwent repair, which was associated with better survival and lower risk of recurrence. This may however not represent the real-world practice. On the other hand, if the native valve is largely disrupted, the choice on the type of prosthesis should consider patient-related factors such as age, compliance to anticoagulants, and life expectancy. In fact, there is currently no evidence of superiority of bioprosthesis or mechanical valves^90^ as they present similar survival and recurrence rates of endocarditis.^91^

Also, the continuing search for the ideal prognostic score for risk stratification in cardiac surgery in IE has been undertaken by several groups,^92 - 95^ although currently no risk score has proven to be superior in IE setting.^96^ Risk stratification before surgery is however crucial and should take into account patient’s clinical status, comorbidities and operative risk.^57^ A decisive role of the multidisciplinary “Endocarditis Team” in timely referral for cardiac surgery and clinical evaluation, especially in left-sided IE cases, has been recognized.^57 , 69^

Post-operative surgical mortality ranges from 6 to 29% in observational series ( Table 2 ). A meta-analysis published by Varela-Barca^79^ in 2019 identified the following factors linked to increased mortality after surgery: age, female, urgent or emergency surgery, previous cardiac surgery, NYHA class ≥ III, cardiogenic shock, prosthetic valve, multivalvular affection, renal failure, perivalvular abscess and *Staphylococcus aureus* infection.

**Table 2:** – Characteristics of observational surgical series on infective endocarditis patients

Study	Country	Study design	Temporal period	N	Native valve (%)/Prosthetic valve (%)	Mortality rate (%)
Jassal et al.^115^	USA	Retrospective	1995-2004	91	85.7/14.3	15.4
Bannay et al.^116^	France	Prospective	1998-2000	240	67.5/32.5	19.4
Lalani et al.^83^	Multicentric	Prospective	2000-2005	720	100/0	12.1
Gaca et al.^95^	USA	Retrospective	2002-2008	19543	NA	8.2
Lalani et al.^86^	Multicentric	Prospective	2000-2005	490	0/100	22
Pang et al.^117^	Singapore	Retrospective	2000-2012	191	92.7/7.3	6.3
Machado et al.^118^	Brazil	Prospective	2003-2010	64	NA	17
Madeira et al.^94^	Portugal	Retrospective	2007-2014	128	75.7/15	16
Olmos et al.^97^	Spain	Prospective	1996-2014	671	60/40	28.6
Pivatto et al.^93^	Brazil	Retrospective	2007-2016	107	-/31	29
Varela el al.^119^	Spain	Retrospective	2002-2016	180	62.6/37.5	26.8
Guiomar et al.^120^	Portugal	Retrospective	2006-2017	145	68/32	13.1

*NA: non-available.*

Although surgical rates tended towards an increase by 7% per decade from 1969 to 2000,^61^ since the beginning of this century, the general trend is towards stability,^4 , 8 , 9 , 61^ even though populational studies conducted in Spain^97^ and the USA^98^ have continuously presented increasing rates. This probably results from recent scientific guidelines, continuous advances in intensive care and surgical management of these patients.

#### Mortality

In-hospital mortality rate varies between countries, from 8 to 40%.^12^ Regarding short-term mortality (up to 30-day follow-up) in populational studies in the last two decades, the rates have ranged between 11-25%, whereas a one-year follow-up revealed a 32% mortality rate ( Table 1 ). A meta-analysis published in 2017^99^ including 25 observational studies, estimated a short- (six months) and long-term follow-up (up to 10 years) mortality rate of 20% and 37% respectively. Fernandez et al.,^33^ Toyoda et al.^11^ and Ilhão Moreira et al.^80^ reported a five-year mortality rate of 52%, 53% and 43%, and Netzer et al.^100^ reported a seven-year mortality rate of 56%.^100^ Although data on long-term follow-up are scarce, current evidence discloses a trend toward a poor prognosis of these patients even if they survive hospitalization for active IE.

Several factors have been linked to increased IE-related mortality. In 2019, a metanalysis^79^ of 16 studies, including 7,484 patients identified female, urgent or emergency surgery, previous cardiac surgery, NYHA class ≥ III, cardiogenic shock, prosthetic valve, multivalvular affection, renal failure, perivalvular abscess and *Staphylococcus aureus* infection as important markers of in-hospital mortality.

Causes of death have been poorly addressed in most series. Fernandez-Hidalgo et al.^33^ described in their prospective observational cohort study of 438 IE patients, an in-hospital mortality rate of 29%, 80% of deaths directly related to IE, whereas the remaining were mostly due to nosocomial infection or major bleeding. Prospective registries^6 , 20^ identified cardiovascular causes, mainly heart failure, and sepsis as main causes of in-hospital mortality in these patients. Long term mortality causes have not been explored.

#### IE prophylaxis

In 2007 in the USA^101^ and 2009 in Europe^57^ indications for antibiotic prophylaxis have been downgraded, with important limitations on the use of antibiotics during dental procedures and withdrawal of antibiotic administration during genitourinary and gastrointestinal procedures. In 2008, the National Institute for Health and Care Excellence (NICE) of the United Kingdom issued guidelines^102^ completely removing all indications on the use of antibiotic prophylaxis for dental and non-dental procedures.

According to European guidelines,^57^ IE antibiotic prophylaxis should be administered to high-risk patients:

1. Patients with a prosthetic valve or with prosthetic material used for cardiac valve repair;
2. Patients with previous IE;
3. Patients with untreated cyanotic CHD and CHD patients with postoperative palliative shunts and conduits, or other prostheses.

In this subpopulation of patients, antibiotic prophylaxis should be used for dental procedures requiring manipulation of the gingival or periapical region of the teeth or perforation of the oral mucosa.

These decisions are not consensual among countries, though. Latin American countries including Brazil remain conservative. IE antibiotic prophylaxis still includes patients with significant valve disease such as degenerative or bicuspid aortic valve, mitral valve prolapse with regurgitation, or rheumatic valve disease. It is also used before genitourinary or gastrointestinal procedures involving mucosa in high-risk patients.^103^

## Incidence and mortality trends

In developed countries such as Denmark, Italy, England, Spain, Germany, Finland, and Netherlands, there has been an increasing trend in the incidence of IE in the last two decades ( Table 3 ). This may be explained by demographic reasons (e.g., aging population), changes in the etiology of valve disease, an increasing number of patients with implanted cardiac devices or prosthesis, an increasing survival of patients with structural and CHD, need for long-term vascular access for different conditions, and advances in prophylaxis measures.

**Table 3:** – Evolution of incidence and/or mortality rates in populational studies on infective endocarditis

Authors/Study	Country	Time period	Increasing incidence trends	Surgical rate trends	Mortality trends
Fonager et al.^1^	Denmark	1980-1997	Yes	NA	Decreasing
Tleyjeh et al.^2^	USA (Olmsted County)	1970-2000	No	Stable	Stable
Sy et al.^7^	Australia (New South Wales)	2000-2006	No	NA	Stable
Fedeli et al.^8^	Italy (Veneto region)	2000-2008	Yes	Stable	Increasing
Bikdeli et al.^109^	USA	1999-2010	No	NA	Stable
Duval et al.^9^	France (Greater Paris, Lorraine, and Rhône-Alpes)	1 yr. survey 1991, 1999 and 2008	No	Increase from 1991-1999. Then stable.	NA
Dayer et al.^111^	England	2000-2013	Yes (more pronounced after 2008)	NA	NA
Pant et al.^112^	USA	2000-2011	Yes (to *Streptococcus* IE after 2007)	Increase 2000-2007/stable 2007-2011	NA
Erichsen et al.^10^	Denmark	1994–2011	Yes (in men and older age)	NA	NA
Keller et al.^62^	Germany	2005-2014	Yes (more pronounced after 2009)	NA	Stable
Toyoda et al.^11^	USA (California and New York State)	1998-2013	No	NA	Stable
Van der Brink et al.^113^	Netherlands	2005-2011	Yes	NA	NA
Cresti et al.^3^	Italy (Grosseto)	1998-2014	Yes	NA	Mild increase (p=0.055)
Olmos et al.^97^	Spain	2003-2014	Yes	Increase	Decrease (0.2%/year)
Ahtela et al.^114^	Finland	2005-2014	Yes	NA	Stable
Shah et al.^65^	Scotland	1990-2014	No (increase in patients >80 years old)	NA	NA
Khan et al.^98^	USA	2002-2016	NA	Increase	A decrease (from 16.7% to 9.7%)

*NA: non-available.*

On the other hand, this trend has not been seen in other countries including France, Australia, Scotland or the United States of America (USA).^2 , 9^ These disparities are probably related to the different sources and definition of cases, and impact timing of improvements in diagnostic methods (imaging, microbiology).

The greater impact of IE prophylaxis on IE incidence in high-risk patients (2007’s American Heart Association^101^ and 2009’s European Society Cardiology^104^ ) has been evaluated in different studies, but uncertainty persists, as Khant et al.^105^ concluded in a metanalysis in 2016. In fact, several authors showed a more pronounced increase in the IE incidence in countries such as United Kingdom, Germany and Netherland ( Table 3 ), whereas DeSimone et al.^106^ and Duval et al.,^9^ from the USA and France respectively, have not detected this trend. Efforts should be made to assess, worldwide, the impact of local guidelines and physician’s compliance on the incidence of IE.

Despite significant advances in the field of diagnosis and management (medical and surgical) of IE, stability is noted regarding in hospital mortality in most populational series. Exceptions were Italy, where an increase was noted,^3 , 8^ and in Denmark,^1^ Spain^97^ and the USA^98^ where a decrease was registered. The ICE^4^ and EURO-ENDO^6^ registries displayed a mildly increased mortality rate of 18% and 17% respectively, compared to the Euro Heart Survey^20^ (13%). Finally, in 2013 a metanalysis^12^ concluded on a decrease in in-hospital mortality from 1960 to 1980 with stability afterwards.

## Challenges and future directions

In the last century, medical and surgical advances allowed for a remarkable improvement in the management and prognosis of IE. Still, physicians face daily challenges when dealing with such patients ( Table 4 ).

**Table 4:** – Current major challenges of infective endocarditis

Main area	Challenges
Prevention	- IE prophylaxis (the use of stricter or broader indications) - clinicians/patients’ awareness
Diagnosis	- criteria - negative blood cultures - prosthesis and cardiac devices – the value of imaging
Medical therapy	- robust evidence (randomized controlled clinical trials) - use of OPAT
Surgery	- robust evidence (randomized controlled clinical trials) - timing - valve repair versus replacement (technical expertise) - in patients with ischemic stroke - in intravenous drug users - in prosthetic endocarditis (always versus occasionally)
Overall management	- heart team versus multidisciplinary IE team - referral centres

*OPAT: Outpatient Parenteral Antimicrobial Therapy. IE: infective endocarditis.*

Prevention should be a priority in national health policies. Patient and physician education campaigns are of crucial importance, and IE prophylaxis, analyzing which patients benefit the most, should be optimized.

Centers of expertise gathering experts in imaging, infectious disease, and cardiology should be established, aiming at better clinical and surgical outcomes. Straightforward communication with non-referral centers should be highly supported. Multimodality imaging protocols should be established, and technological improvements researched. The need to reduce hospital length of stay with the establishment of well-trained, outpatient teams and educated patients that would allow for OPAT, whenever feasible, must also be endorsed by institutions.

Evidence-based investigation is still quite exceptional and globally heterogeneous. In fact, most of our data were obtained from registries, populational studies and single/multicenter experiences in middle-to-high income countries, allowing for a non-neglectable selection bias when considering the worldwide condition. Randomized controlled trials should be performed to provide further evidence specifically regarding timing of surgery, antibiotic schemes, the effect of adjunctive medical therapy during the active treatment of IE or use of prosthetic material less predisposed to bacteria adhesion.

As a final comment, the use of artificial intelligence networks that are currently being built in high-volume centers^107^ will allow an accurate estimation of the risk of complications and the ideal surgical timing, ultimately improving patient’s prognosis.
